# Portable System for Real-Time Detection of Stress Level

**DOI:** 10.3390/s18082504

**Published:** 2018-08-01

**Authors:** Jesus Minguillon, Eduardo Perez, Miguel Angel Lopez-Gordo, Francisco Pelayo, Maria Jose Sanchez-Carrion

**Affiliations:** 1 Department of Computer Architecture and Technology, University of Granada, 18014 Granada, Spain; fpelayo@ugr.es; 2 Research Centre for Information and Communications Technologies (CITIC), University of Granada, 18014 Granada, Spain; edu@ugr.es (E.P.); malg@ugr.es (M.A.L.-G.); 3 Department of Signal Theory, Telematics and Communications, University of Granada, 18014 Granada, Spain; 4 Nicolo Association, 18194 Churriana de la Vega, Spain; 5 School for Special Education San Rafael, 18001 Granada, Spain; MariaJose.SanchezC@sjd.es

**Keywords:** stress, biosignal, EEG, ECG, EMG, GSR, real-time, healthcare, e-Health, m-Health

## Abstract

Currently, mental stress is a major problem in our society. It is related to a wide variety of diseases and is mainly caused by daily-life factors. The use of mobile technology for healthcare purposes has dramatically increased during the last few years. In particular, for out-of-lab stress detection, a considerable number of biosignal-based methods and systems have been proposed. However, these approaches have not matured yet into applications that are reliable and useful enough to significantly improve people’s quality of life. Further research is needed. In this paper, we propose a portable system for real-time detection of stress based on multiple biosignals such as electroencephalography, electrocardiography, electromyography, and galvanic skin response. In order to validate our system, we conducted a study using a previously published and well-established methodology. In our study, ten subjects were stressed and then relaxed while their biosignals were simultaneously recorded with the portable system. The results show that our system can classify three levels of stress (stress, relax, and neutral) with a resolution of a few seconds and 86% accuracy. This suggests that the proposed system could have a relevant impact on people’s lives. It can be used to prevent stress episodes in many situations of everyday life such as work, school, and home.

## 1. Introduction

Stress is a major concern in our modern society. According to the 2014 report of the American Psychological Association, most of U.S. population regularly experience physical (77%) or psychological (73%) symptoms caused by stress, the main ones being fatigue (51%), headache (44%), and upset stomach (34%). In addition, chronic stress has been proved to facilitate the development of diseases due to weakening of the immune system [1]. All this adds up to important costs in terms of people’s quality of life and loss of money (USD 300 billion of annual cost to employers in stress related health care and missed work). According to the same report, the top causes of stress in the US are job pressure, money, health, and relationships. Therefore, stress is mainly caused by everyday-life factors. Thus, it is crucial to develop reliable and usable systems for real-time detection of stress level in people’s daily life.

New technologies have attempted to improve people’s quality of life in the last few years [2]. The development of pervasive and ubiquitous systems and applications has led us into modern terms such as e-Health and m-Health. These two concepts encompass information, communication, and mobile technologies for healthcare purposes. e-Health has shown a relevant impact on the quality and safety of healthcare [3]. For example, facilitating the communications between institutions [4], incrementing patient engagement to treatment [5], promoting physical activity in older adults [6], and improving mental health services for trauma survivors [7]. m-Health, for its part, has shown its effectiveness in multiple scopes, such as monitoring health in elderly people [8], promoting early diagnosis of cardiovascular diseases [9], differentiating between Parkinson’s disease and essential tremor diagnosis [10], improving hypertension control in stroke survivors [11], and supporting recovery from drug addiction [12].

Regarding the stress detection, methods and systems based on biosignal analysis are under study. These objective approaches are usually more powerful than self-perception of stress level [13]. For example, some patterns extracted from electrocardiography (ECG) such as heart rate or heart rate variability have been related to mental stress [14,15,16,17,18,19]. The activity of some muscles such as the trapezius has been proved to be connected with stress [20,21,22,23]. The muscle activity can be measured by electromyography (EMG). Other studies have demonstrated the relationship between stress and certain brain rhythms measured by electroencephalography (EEG) [24,25,26,27,28,29,30,31]. The skin conductance has also been correlated with stress [32,33,34]. This parameter can be measured using galvanic skin response (GSR) sensors. All this knowledge has been used by many researchers to propose portable systems for assessment and detection of mental stress. These systems usually combine multiple biosignals. Examples include wearable assessment of mental stress of combatants [35], wristband sensor to measure stress level for people with dementia [36], and stress detection in drivers [37,38,39]. In short, much useful work has been done. Nevertheless, beyond the commercial gadgets, ambulatory stress-monitoring has not matured yet in applications that are reliable and valid enough to convincingly improve people’s health and quality of life. Further research is needed in this field aimed at tackling such an important and serious problem.

In this work, we present and validate a portable system for real-time detection of stress level, based on the RABio w8 (real-time acquisition of biosignals, wireless, eight channels) system. We have designed and implemented both hardware and software in our laboratory. The hardware is made of portable, wireless, and low-cost electronics. The software is composed by an application programming interface (API) and a graphical user interface (GUI). We conducted a study to validate our system using proven and well-established methodology to induce different levels of stress. Our results demonstrate the potential application of our system as a useful tool for ubiquitous stress monitoring, detection, and prevention.

## 2. Materials and Methods

### 2.1. Description of the System

As mentioned before, the portable system for real-time detection of stress level presented in this work is based on the RABio w8 system. RABio w8 is a portable, wireless, low-cost hardware–software system for the acquisition and processing of multiple biosignals such as EEG, ECG, and EMG. It has been used in previous works [40].

**Figure 1:**
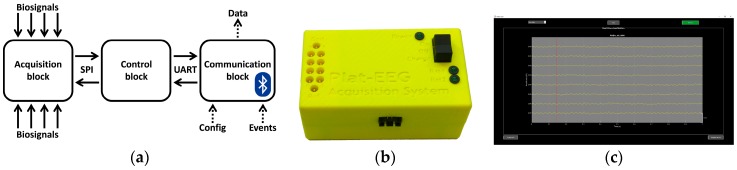
RABio w8 system: (**a**) Diagram of the electronics; (**b**) Picture of the hardware; (**c**) Screenshot of the graphical user interface (GUI).

The electronics of RABio w8 is composed of three blocks (see Figure 1a): acquisition block, control block, and communication block. The acquisition block uses advanced integrated circuits for biosignal acquisition from the ADS family of Texas Instruments (Dallas, TX, USA). This block is in charge of the amplification and the analogue-digital conversion of eight simultaneous channels, up to 1000 samples per second with 24-bit sample resolution. The gain factor of every single channel and the sampling rate are configurable. This block interacts with the control block through a serial peripheral interface (SPI). The control block uses a microcontroller from Microchip Technology (Chandler, AZ, USA) to receive, synchronize, format, and send the data frames from the first block to the communication block through a universal asynchronous receiver–transmitter (UART) port. Finally, the communication block is responsible for the wireless communication with the software of RABio w8 via Bluetooth. All the electronics are powered by high-autonomy lithium polymer rechargeable batteries and contained in a 3D printed plastic casing (see Figure 1b).

The software of RABio w8 is composed by an application programming interface and a graphical user interface. The API is a dynamic-link library of Windows OS coded in C/C++. It allows one to receive data frames from the electronics of RABio w8, as well as to configure the acquisition parameters (i.e., channels gain and sampling rate) and to send event markers via Bluetooth. The GUI of RABio w8 (see Figure 1c) is coded in Matlab from The Mathworks (Natick, MA, USA). The GUI uses the functions provided by the API to allow the user to visualize process and record the signals acquired by the electronics in real-time. Configuration of acquisition parameters and event marking is also available for the user.

**Figure 2:**
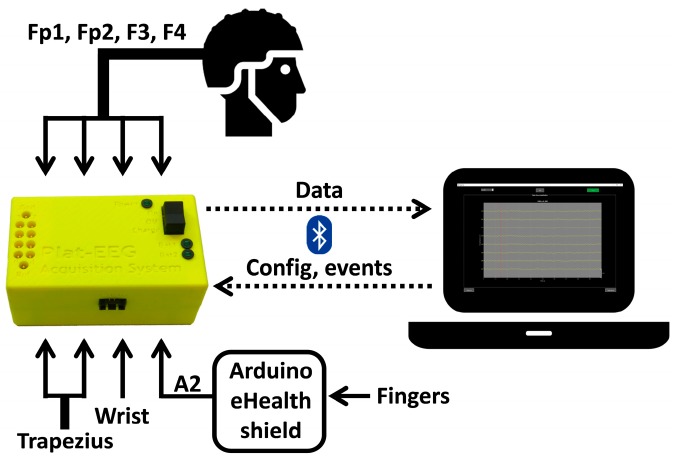
Diagram of the full portable system for real-time detection of stress level. The system is composed by the RABio w8, multiple biosignal sensors placed at head, trapezius, wrist and fingers, the Arduino e-Health platform, and a laptop.

The full portable system for real-time detection of stress level (see Figure 2) consists of multiple biosignal sensors (EEG, ECG, EMG, and GSR electrodes), the RABio w8 system, a laptop, and the e-Health sensor platform of Arduino. The EEG, ECG, and EMG electrodes are directly attached to input channels of the RABio w8 hardware. The GSR electrodes are attached to the e-Health shield. This shield is powered by an Arduino board and provides skin conductance measurements. The measured values are sent to the RABio w8 hardware by connecting the analogue output of the shield (A2) to an input channel of RABio w8. The laptop is in charge of visualizing, processing, and recording the acquired biosignals using the API and the GUI of RABio w8.

For the purpose of this work (i.e., presentation and validation of our system), a laptop was used. However, in a final version, we propose the cloud-computing of biosignals with real-time biofeedback presented in mobile devices such as tablets or smartphones. Also, a more wearable version of the EEG cap embedding the whole electronics is feasible and under development.

### 2.2. Experimental Procedure

We conducted a study in order to validate our system, following the well-established methodology of previous published stress studies [28,31]. Ten healthy volunteers were involved in the study (five male, five female, age range of 18–23 years, mean age of 20 ± 2 years, all of them novice in stress-related experiments). The recruitment process started one month prior to the beginning of the study by means of informative emails. The participants were instructed to avoid stimulants or relaxant substances in the 3 h prior to the experiment. They were not paid for their participation. They were provided with the experiment’s information sheet and the informed consent, both of which were approved by the Bioethics Committee of the University of Granada.

**Figure 3:**
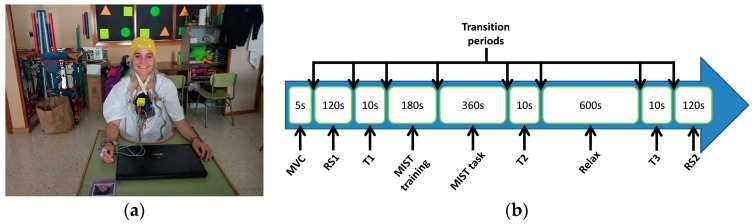
(**a**) Picture of one participant ready for the experiment after preparation; (**b**) timeline of the experiment. Duration of each part is in seconds (s). The total duration was around 30 min, including the transition periods (see text for details). MVC—maximum voluntary contraction; RS—resting state block; MIST—Montreal imaging stress task.

The participants were prepared by the research staff after they read, understood, and signed the informed consent (see Figure 3a). They wore white hospital clothes during the experiment. Four EEG electrodes were placed at Fp1, Fp2, F3, and F4 positions of the 10–20 International System using an EEG cap. These positions have been successfully used in stress studies [24,25,27,28,31]. One ECG electrode was placed on the wrist of the non-dominant hand. Two EMG electrodes were placed on the trapezius muscle of the non-dominant-hand side, with an inter-electrode distance of 25 mm. The activity of the trapezius has been related to stress in several published studies [20,21,22,23]. Two GSR electrodes were placed on the index and the middle fingers of the non-dominant hand [32,33]. All the electrodes were referenced and grounded to the ear lobe of the dominant-hand side. All the electrode impedances were below 30 KΩ. The EEG, ECG, and EMG electrodes were directly attached to the input channels 0–6 of RABio w8. The GSR electrodes were attached to the Arduino e-Health shield and the analogue output A2 was connected to the input channel 7 of RABio w8, as described in Section 2.1.

Once the participants were prepared, they were instructed to avoid unnecessary movements during the experiment in order to prevent severe artifacts in recordings. They performed a maximum voluntary contraction (MVC) of the trapezius during 5 s and a resting state block (RS1) with closed eyes during 2 min. Afterwards, they were asked about their self-perceived level of stress (T1). The question was posed in Spanish. The English translation is: *If 0 is the minimum level and 4 is the maximum level, what is your level of stress?* The participants then started a stress session. In that session, they performed the Montreal imaging stress task (MIST), a proven methodology that induces psychosocial stress in people [41]. Despite that there are other well-described stress methods such as the variants of the Trier social stress task [42], the MIST has been used in a considerable number of stress-related works [28,31,41,43,44,45]. It was classified as well-described stress method by a recent review [46]. The MIST consists of two parts: training and task. In the training part, the participant is asked to solve arithmetic operations without time limit per operation. The difficulty level of the operations randomly varies (five levels). In the task part, the participant has to solve arithmetic operations with time limit. The time limit adapts according to the number of consecutive wrong and right answers. This enforces a range of 20–45% success ratio, while the participant is asked to achieve about 80–90%. The participant is periodically reminded of the relevance of achieving the goal. Detailed information of this protocol can be found in the literature [41]. In our study, after a training of 3 min, the task lasted 6 min. During that session, the participants were seated on a comfortable chair within a classroom while they were using the touchpad of a laptop to play a Matlab-based GUI of the MIST. This GUI was developed by us and further details including screenshots can be found in the literature [28]. After the stress session, the question about the self-perceived level of stress was asked again (T2).

Immediately after the stress session, the participants started a relaxing session. During that session, they stayed laid (resting state with opened eyes) down in a blue-lighted room for 10 min. Blue light was recently proven to accelerate the relaxation process after the MIST in comparison with conventional white light [31]. In this work, the same room and light were used. Once again, the question about the self-perceived stress level was asked at the end of the relaxing session (T3). Finally, a new resting state block (RS2) with closed eyes was performed for 2 min. The timeline of the experiment is shown in Figure 3b.

All the biosignals (raw data) were recorded during the whole experiment at 1000 samples per second with amplification gain of 3 for EEG channels and 1 for the others. All the events (e.g., start of stress session, end of stress session, etc.) were marked in the data. For the aim of this work (i.e., presentation and validation of our system), the biosignals were processed and analyzed offline. The real-time capability of our system is discussed in Section 4.2.

### 2.3. Signal Processing

#### 2.3.1. EEG

EEG data were zero-phase bandpass filtered (1–48 Hz) with a fourth-order Butterworth infinite impulse response (IIR) filter. Data corresponding to regions of interest (i.e., central minute of each resting-state block, stress session, and relaxing session) were segmented into two-second epochs (no overlap of consecutive epochs). Detrending and z-score normalization was applied to each epoch. The power in theta–alpha (4–13 Hz) and gamma (25–45 Hz) bands was estimated for each channel and then averaged across channels. The average relative gamma (RG) was computed for every single epoch as the power ratio between the average gamma power and the average theta–alpha power. The RG is a stress marker used in emotion and stress studies [28,29,30,31]. The following equation defines the RG:

RG = AvPower (25–45 Hz)/AvPower (4–13 Hz)(1)

#### 2.3.2. ECG

ECG data were zero-phase bandpass filtered (16–24 Hz) with a second-order Butterworth IIR filter in order to enhance the R-peak of the QRS complex. Data corresponding to parts of interest were segmented into 10-s epochs (no overlap of consecutive epochs). The average heart rate (HR) in beats per minute was computed for each epoch by means of the average R–R-interval length. It was not possible to compute the HR using two-second epochs. The set of HR values corresponding to 10-s epochs was interpolated using a spline to obtain values corresponding to two-second epochs. The HR is also a stress marker widely used in stress studies [14,15,16,17,18,19]. The following equation defines the HR:

HR (bpm) = 60/AvRR(2)

#### 2.3.3. EMG

EMG data were zero-phase bandpass filtered (1–350 Hz) with a second-order Butterworth IIR filter. In order to obtain differential EMG data, data corresponding to the electrode further from the backbone was subtracted from data corresponding to the electrode closer to the backbone. Differential data corresponding to parts of interest were segmented into two-second epochs (no overlap of consecutive epochs). The average trapezius activity (TA) was computed for each epoch as the ratio between the root mean square (RMS) value in the epoch and the RMS value in the MVC test. As in the case of RG and HR, the TA is also a stress marker used in several stress studies [20,21,22,23]. The following equation defines the TA:

TA = RMS (epoch)/RMS (MCV test)(3)

#### 2.3.4. GSR

GSR data corresponding to parts of interest were directly segmented into two-second epochs (no overlap of consecutive epochs). The average skin conductance (SC) in Siemens was computed for each epoch by using the equation provided by the Arduino e-Health platform tutorial. The SC is one of the most used stress markers in literature [32,33,34]. The following equation defines the SC:

SC = 2 × (AvVoltage − 0.5)/100,000(4)

### 2.4. Statistical Analysis

The grand-average across subjects of the time evolution of processed stress markers in the regions of interests (i.e., set of values of RG, HR, TA, and SC corresponding to two-second epochs) was computed. For a better visualization, individual data were z-scored and smoothed using a moving average filter (10 samples) before the computation of the grand-average. The grand-average of the self-perceived stress level (SPSL) at the three test points (i.e., T1, T2, and T3) was also computed. A paired-sample t-test was applied in order to assess if the stress markers and the SPSL significantly differ (p-value < α with α = 0.05) at different time periods. In particular, T1, T2, and T3 were compared for the SPSL. The last 30 s of the first resting state block, the last 30 s of the stress session, and the second-to-last 30 s of the relaxing session were compared for the stress markers. Finally, the Pearson’s correlation coefficient (PCC) between grand-averaged stress markers and the corresponding 95% confidence interval (CI) was calculated.

### 2.5. Three-Level Stress Classification

A linear discriminant analysis (LDA) was performed to detect the level of stress using the processed stress markers (i.e., RG, HR, TA, and SC) as features. Three classes (i.e., levels of stress) were defined: stress, relax, and neutral. The values corresponding to the two-second epochs of the minutes 7–8 of the stress session were labeled as stress. These epochs corresponds to the period of maximum stress. The values corresponding to the two-second epochs of the minutes 2–3 of the relaxing session were labeled as relax. These epochs corresponds to the period of minimum stress. The values corresponding to the two-second epochs of the central minute of each resting-state block were labeled as neutral. These epochs corresponds to the periods of baseline stress level. Therefore, 60 observations (120 s with two-second-epoch values) per class were used. A leave-one-out cross validation (LOOCV) was performed for the three-class LDA. That is, for all the observations, 179 out of 180 observations were used in training to classify the remaining observation. In addition to the leave-one-epoch-out cross validation, a leave-one-subject-out cross validation was conducted. That is, for all the subjects, the epochs of one subject were classified using the epochs of the remaining subjects as training data. The classification accuracy or probability of success (p_a_) in stress level detection was computed as the ratio between the number of successfully classified observations and the total number of observations (i.e., n = 180). The 95% CI was also estimated as follows:

CI = p_a_ ± 1.96 × sqrt(p_a_ × (1 − p_a_)/n) (5)

## 3. Results

### 3.1. Time Evolution of Biosignal-Based Markers

**Figure 4:**
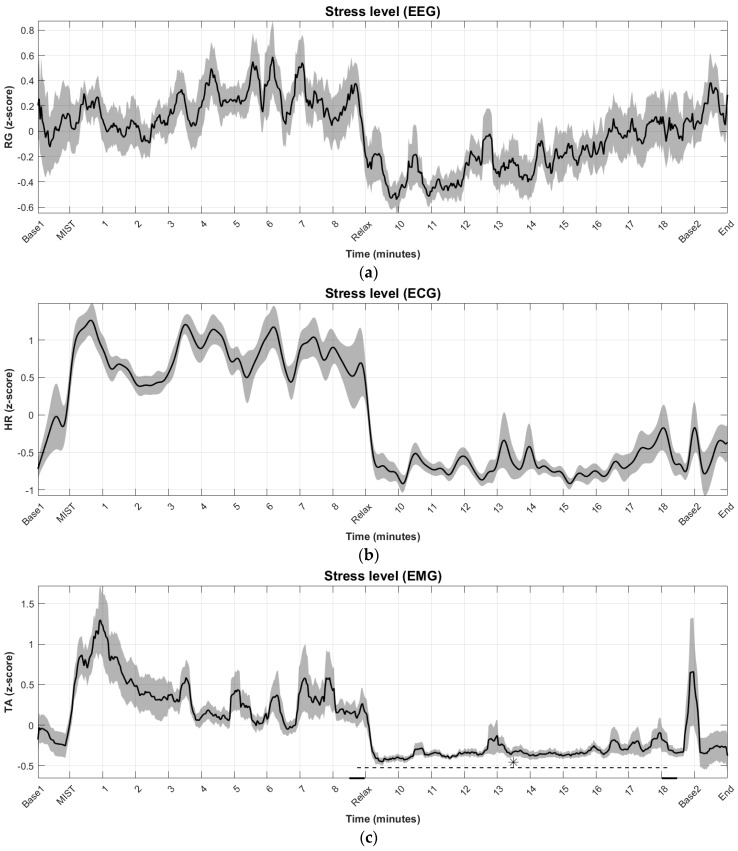

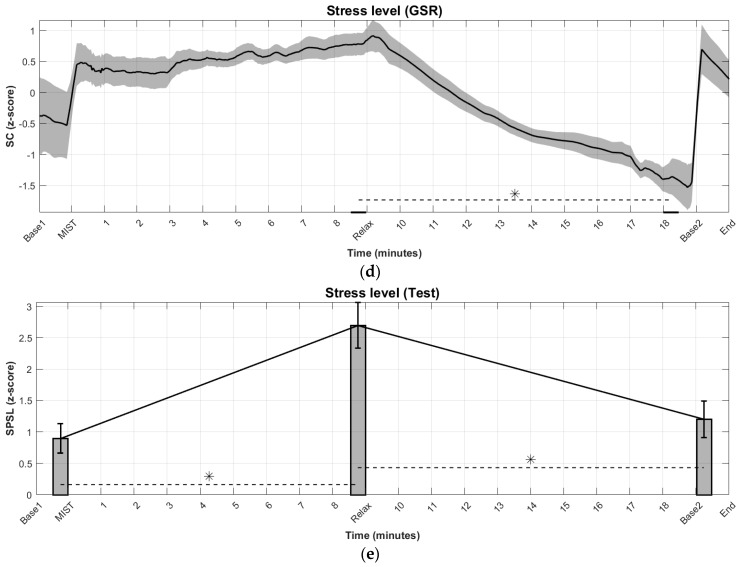
Grand-average across subjects of the time evolution of processed stress markers in the regions of interests. Base1 and Base2 correspond to the central minutes of resting state blocks RS1 and RS2, respectively. MIST indicates the beginning of the stress session (3 min of training and 6 min of task). Relax indicates the beginning of the relaxing session. Shades behind the plots and error bars indicate the standard error of the mean (SEM): (**a**) relative gamma (RG) estimated from electroencephalography (EEG) data; (**b**) average heart rate (HR) estimated from electrocardiography (ECG) data; (**c**) trapezius activity (TA) estimated from electromyography (EMG) data. Asterisk indicates statistically significant difference (*p*-value < 0.05) in average TA between the last 30 s of the stress session and the second-to-last 30 s of the relaxing session; (**d**) skin conductance (SC) estimated from galvanic skin response (GSR) data. Asterisk indicates statistically significant difference (*p*-value < 0.05) in average SC between the last 30 s of the stress session and the second-to-last 30 s of the relaxing session; (**e**) self-perceived stress level (SPSL) obtained from questions at T1, T2, and T3 points. X-axis only comprises regions of interests and T1, T2, and T2 would actually be located before the stress session (i.e., just before MIST), after the stress session (i.e., just after minute 9), and after the relaxing session (i.e., just after minute 19), respectively. Asterisks indicate statistically significant difference (*p*-value < 0.05) in SPSL between the T1–T2 and between T2–T3.

Figure 4a–d show the grand-average across subjects of the time evolution of processed stress markers in the regions of interests. Figure 4e also shows the grand-average of the SPSL at the three test points (i.e., T1, T2 and T3).

In addition, the Pearson’s correlation coefficient (PCC) between stress markers and the corresponding 95% confidence interval is reported in Table 1.

### 3.2. Stress Level Detection

The classification accuracy or probability of success (p_a_) in detection of stress level (stress, relax, and neutral) and the 95% confidence interval are reported in this section. In particular, Table 2 shows these values when using one stress marker as feature for the LDA classifier. Table 3 shows the same statistics when using two stress markers as features. Table 4 shows the same when using three or all the stress markers. Finally, Table 5 shows the same as Table 4, but using a leave-one-subject-out cross validation instead of leave-one-epoch-out. In these four tables, main values indicate the pa and error values indicate the 95% CI. Last row indicates the mean and the standard deviation of the mean. All the values are expressed in percentage.

## 4. Discussion

### 4.1. Stress and Biosignals

At first sight, the time evolution of all the stress markers indicates agreement with the self-perception of stress level. This is partially supported by the statistical tests (see Figure 4). The MIST causes a significant increase in self-perceived stress level and the relaxing session causes a significant decrease. However, only two stress markers presented significant differences in stress level at different time periods. These are the TA and the SC. The significant differences were only between the end of the stress session (maximum level of stress) and the end of the relax session (minimum or very low level of stress). The other two markers (i.e., RG and HR) did not present significant differences despite the noticeable changes. All the markers reflect that the increase in stress level is gradual. This behavior has been reported in previous literature [28,31]. However, there are some visible differences between markers. In particular, the RG, the HR, and the TA indicates that the minimum level of stress is quickly achieved once the relaxing session starts (less than 1 min from the beginning of this session), while the SC denotes a gradual decrease. This is due to the fact that the sweating process is fast, while the reabsorption process is slow in comparison with other physiological responses. Accordingly, the SC has a drawback in terms of time of response. In addition, the RG is the only marker that reflects a gradual increase in stress level during the relaxing session. This fits with results reported in previous literature and may be caused by boredom of the participants during this part of the experiment [28,31]. The other markers indicate a more rapid increase at the end of the relaxing session. In this regard, the RG has an advantage in terms of response time. In other words, the boredom may have an immediate effect on EEG, while it may have a delayed effect on the other biosignals.

Regarding the PCC between stress markers, all of them are generally correlated (see Table 1). The one that correlates the most with the others is the HR (72.96% with RG, 83.38% with TA, and 63.27% with SC). The SC is the least correlated marker (32.93% with RG, 63.27% and 46.32% with TA). This is due to the response time discussed in the previous paragraph and to the fact that the GSR is the least noisy biosignal (see Figure 4). The ECG is the second least noisy biosignal. This suggests that ECG and GSR are the more appropriate biosignals in the presence of artifacts. Nevertheless, the stress markers extracted from these two biosignals and from the EMG can be misrepresented by physical activity (e.g., physical activity may increase the HR even without being stressed). In this respect, the RG is advantageous.

### 4.2. Real-Time Detection of Stress Level

The results of the three-class LDA with leave-out-epoch-out cross validation (see Table 1, Table 2, Table 3 and Table 4) indicate that the more biosignals (thus more stress markers) that are combined, the higher probability of successful detection of stress level (i.e., accuracy). With single markers, the probabilities are 50%, 72%, 42%, and 60% for RG, HR, TA, and SC, respectively. With two markers, probabilities are 78% (RG–HR), 64% (RG–TA), 67% (RG–SC), 77% (HR–TA), 79% (HR–SC), and 73% (TA–SC). However, the probabilities increase up to 82% (RG–HR–TA), 82% (RG–HR–SC), 75% (RG–TA–SC) and 84% (HR–TA–SC). There is no relevant improvement by adding the RG to the trio HR–TA–SC (86%). By using three or all the markers, the results overcome accuracies reported in previous studies of biosignal-based systems for stress detection and measurement [32,33,35,39,47]. Nevertheless, this is not meaningful because the cited studies were neither carried out in the same experimental conditions, with comparable number of subjects, nor were they conducted with a similar methodology. In reference to the results of the leave-one-subject-out cross validation (see Table 5), the probabilities of success are generally close to the chance level (i.e., 33%), taking into account the confidence intervals. This indicates that the system needs to be calibrated for every single subject. This was expected as stress markers and thresholds may vary across subjects. Regarding the optimal combination of markers, it depends on the particular conditions in which the stress has to be detected (e.g., response time and external factors). For example, in this work, the use of EEG signals does not optimize the results in terms of accuracy. The EEG has a distinct set of advantages and limitations. Among the advantages, as cited in Section 4.1, the markers based on brain activity (e.g., the RG) present a shorter response time and are less susceptible to physical activity. Additionally, the EEG provides powerful endogenous and cognitive information such as attention [48,49,50] that can be useful in certain scenarios. Regarding the limitations, the use of EEG provides a number of technical challenges such as additional sensors (thus less portability) or higher computational complexity. In order to overcome some of these limitations, we are developing a more wearable version of the EEG cap embedding the whole electronics and based on dry electrodes [51]. Our results demonstrate the reliability of our system in the detection of three levels of stress with a resolution of a few seconds. Still, the results could improve by extracting more features (i.e., stress markers) from biosignals [38] and by using more powerful classifiers such as artificial neural networks [52,53]. For the aim of this work, biosignals were processed offline. We used two-second epochs of data with a low-cost preprocessing, feature extraction, and classification in terms of computation time. This provides our system with real-time capability. In addition, for the sake of simplicity, the participants of the study were instructed to avoid unnecessary movements during the experiment in order to prevent severe artifacts in recordings. This is an unrealistic scenario. For the use of the proposed system in daily-life scenarios, advanced processing for artifact removal should be included [2]. Based on the accuracies obtained in this work, we expect that our system can still work in hostile environments by adding the artifact removal part.

## 5. Conclusions

In this work, we have proposed a portable system for real-time detection of stress level. We have presented the methodology and the results of a study aimed at validating the system. In the study, ten volunteers were stressed and then relaxed using well-established methods, while their biosignals were recorded. Our portable system can simultaneously record and process four types of biosignals (i.e., EEG, ECG, EMG, and GSR) in real-time, thereby enabling the detection of three levels of stress very accurately (86%). The system has some limitations that have been discussed (e.g., portability and performance under artifacts). In order to overcome them, we are working on a final version in which the biosignals are cloud-computed, including the needed processing for artifact removal. The real-time biofeedback (i.e., 2 s plus the computation time) will be presented in mobile devices such as tablets or smartphones. Moreover, a more wearable version of the EEG cap embedding the whole electronics is feasible and under development. Having overcome the cited limitations, our system could be used as a reliable tool for real-time stress monitoring, detection, and prevention in daily life. For example, prevention of job stress in periods of high level of work intensity, stress monitoring in children at school, or discovery of new stressors through stress detection in the domestic environment. All of this has a relevant impact on society as stress is a major problem nowadays and this system could substantially improve people’s health and quality of life.

## Figures and Tables

**Table 1:** Pearson’s correlation coefficient (PCC) between processed stress markers and the corresponding lower (CI low) and upper (CI up) bounds for a 95% confidence interval (CI). RG—relative gamma; HR—average heart rate; TA—trapezius activity; SC—skin conductance.

Pair	PCC	CI Low	CI Up
RG, HR	0.7296	0.6909	0.7642
RG, TA	0.5753	0.5206	0.6253
RG, SC	0.3293	0.2579	0.3972
HR, TA	0.8338	0.8083	0.8561
HR, SC	0.6327	0.5834	0.6773
TA, SC	0.4632	0.3995	0.5224

**Table 2:** Probability of successful detection of stress level using ones stress marker as feature.

Participant	RG	HR	TA	SC
1	72 ± 7	74 ± 6	31 ± 7	49 ± 7
2	61 ± 7	57 ± 7	28 ± 7	69 ± 7
3	61 ± 7	45 ± 7	29 ± 7	84 ± 5
4	51 ± 7	60 ± 7	61 ± 7	51 ± 7
5	28 ± 7	93 ± 4	22 ± 6	69 ± 7
6	44 ± 7	94 ± 3	45 ± 7	61 ± 7
7	47 ± 7	82 ± 6	66 ± 7	60 ± 7
8	33 ± 7	77 ± 6	21 ± 6	61 ± 7
9	67 ± 7	77 ± 6	52 ± 7	18 ± 6
10	33 ± 7	62 ± 7	62 ± 7	76 ± 6
Mean ± Std	50 ± 15	72 ± 16	42 ± 18	60 ± 18

**Table 3:** Probability of successful detection of stress level using two stress markers as features.

Participant	RG, HR	RG, TA	RG, SC	HR, TA	HR, SC	TA, SC
1	76 ± 6	83 ± 6	69 ± 7	86 ± 5	71 ± 7	64 ± 7
2	73 ± 6	82 ± 6	73 ± 6	61 ± 7	70 ± 7	78 ± 6
3	77 ± 6	60 ± 7	81 ± 6	52 ± 7	92 ± 4	90 ± 4
4	59 ± 7	64 ± 7	72 ± 7	70 ± 7	68 ± 7	87 ± 5
5	92 ± 4	46 ± 7	54 ± 7	93 ± 4	84 ± 5	76 ± 6
6	94 ± 3	69 ± 7	64 ± 7	93 ± 4	96 ± 3	71 ± 7
7	84 ± 5	66 ± 7	64 ± 7	86 ± 5	86 ± 5	66 ± 7
8	74 ± 6	48 ± 7	61 ± 7	76 ± 6	71 ± 7	64 ± 7
9	82 ± 6	72 ± 7	64 ± 7	78 ± 6	73 ± 6	49 ± 7
10	67 ± 7	54 ± 7	67 ± 7	73 ± 6	77 ± 6	81 ± 6
Mean ± Std	78 ± 11	64 ± 13	67 ± 7	77 ± 14	79 ± 10	73 ± 12

**Table 4:** Probability of successful detection of stress level using three or all the stress markers as features.

Participant	RG, HR, TA	RG, HR, SC	RG, TA, SC	HR, TA, SC	RG, HR, TA, SC
1	91 ± 4	79 ± 6	84 ± 5	92 ± 4	92 ± 4
2	82 ± 6	78 ± 6	83 ± 6	75 ± 6	82 ± 6
3	77 ± 6	93 ± 4	82 ± 6	92 ± 4	93 ± 4
4	68 ± 7	69 ± 7	78 ± 6	82 ± 6	83 ± 6
5	93 ± 4	84 ± 5	73 ± 7	84 ± 5	84 ± 5
6	93 ± 4	97 ± 3	72 ± 7	98 ± 2	98 ± 2
7	86 ± 5	87 ± 5	67 ± 7	89 ± 4	90 ± 4
8	74 ± 6	75 ± 6	64 ± 7	71 ± 7	74 ± 6
9	81 ± 6	80 ± 6	67 ± 7	76 ± 6	81 ± 6
10	72 ± 7	77 ± 6	79 ± 6	79 ± 6	78 ± 6
Mean ± Std	82 ± 9	82 ± 8	75 ± 7	84 ± 9	86 ± 8

**Table 5:** Probability of successful detection of stress level using three or all the stress markers as features for the leave one-subject-out cross validation.

Participant	RG, HR, TA	RG, HR, SC	RG, TA, SC	HR, TA, SC	RG, HR, TA, SC
1	33 ± 7	33 ± 7	36 ± 7	33 ± 7	33 ± 7
2	67 ± 7	37 ± 7	58 ± 7	64 ± 7	65 ± 7
3	33 ± 7	41 ± 7	36 ± 7	33 ± 7	36 ± 7
4	47 ± 7	36 ± 7	33 ± 7	49 ± 7	34 ± 7
5	66 ± 7	41 ± 7	37 ± 7	64 ± 7	38 ± 7
6	36 ± 7	34 ± 7	33 ± 7	34 ± 7	34 ± 7
7	33 ± 7	33 ± 7	39 ± 7	33 ± 7	33 ± 7
8	34 ± 7	53 ± 7	51 ± 7	54 ± 7	54 ± 7
9	41 ± 7	60 ± 7	56 ± 7	51 ± 7	66 ± 7
10	48 ± 7	48 ± 7	48 ± 7	36 ± 7	42 ± 7
Mean ± Std	44 ± 13	42 ± 9	43 ± 10	45 ± 13	44 ± 13
